# Contributions of carbon source, crop cultivation, and chemical property on microbial community assemblage in soil subjected to reductive disinfestation

**DOI:** 10.3389/fmicb.2023.1146207

**Published:** 2023-02-24

**Authors:** Weijing Zhu, Xiaolin Lu, Chunlai Hong, Leidong Hong, Fengxiang Zhu, Shuo Zhang, Yanlai Yao

**Affiliations:** ^1^State Key Laboratory for Managing Biotic and Chemical Threats to the Quality and Safety of Agro-Products, Institute of Environment, Resource, Soil and Fertilizer, Zhejiang Academy of Agricultural Sciences, Hangzhou, China; ^2^Ningbo Agricultural and Rural Green Development Center, Ningbo, China

**Keywords:** reductive soil disinfestation, carbon source, cultivation, chemical property, microbial communities, *Fusarium*, yield

## Abstract

In agricultural practice, reductive soil disinfestation (RSD) is an effective method for eliminating soil-borne pathogens that depends heavily on carbon source. However, knowledge regarding the assembly of soil microbial communities in RDS-treated soils amended with different carbon sources after continuous crop cultivation is still not well-characterized. RSD treatments were performed on greenhouse soil with six different carbon sources (ethanol, glucose, alfalfa, wheat bran, rice bran, and sugarcane residue), which have different C:N ratios (Org C/N) and easily oxidized carbon contents (Org EOC). After RSD, two consecutive seasons of pepper pot experiments were conducted. Then, the effects of carbon source property, crop cultivation, and soil chemical property on soil microbial community reestablishment, pathogen reproduction, and crop performance were investigated in the RSD-cropping system. Variation partition analysis indicated that carbon source property, crop cultivation, and soil chemical property explained 66.2 and 39.0% of bacterial and fungal community variation, respectively. Specifically, Mantel tests showed that Org C/N, crop cultivation, soil available phosphorus and potassium were the most important factors shaping bacterial community composition, while Org C/N, Org EOC, and crop cultivation were the most important factors shaping fungal community composition. After two planting seasons, the number of cultivable *Fusarium* was positively correlated with Org EOC, and negatively correlated with soil total organic carbon, Fungal Chao1, and Fungal PC1. Crop yield of complex-carbon soils (Al, Wh, Ri and Su) was negatively affected by Org C/N after the first season, and it was highest in Al, and lower in Et and Su after the second season. Overall, Org EOC and Org C/N of carbon source were vitally important for soil microbe reestablishment, *Fusarium* reproduction and crop performance. Our findings further broaden the important role of carbon source in the RSD-cropping system, and provide a theoretical basis for organic carbon selection in RSD practice.

## 1. Introduction

Reductive soil disinfestation (RSD), also called anaerobic soil disinfestation (Shinmura, 2000) or biological soil disinfestation (Blok et al., 2000), involves adding easily degradable organics to the soil, saturation with water, and sealing with a plastic film to create a strongly reducing and anaerobic environment (Momma et al., 2013). An extensive range of pathogens can be controlled with RSD, such as *Verticillium dahlia*, *Fusarium oxysporum* f. sp. *Fragariae*, *Fusarium oxysporum* f. sp. *Lycopersici*, *Phytophthora nicotianae*, *Rhizoctonia solani*, as well as plant-parasitic nematodes (Korthals et al., 2014; Huang et al., 2016; Serrano-Perez et al., 2017; Henry et al., 2020; Lee et al., 2020; Zavatta et al., 2021). As an alternative to chemical fumigation for soil-borne pathogen control, RSD has been widely applied in solving continuous cropping problems of various cash crops, such as watermelon, strawberry, tomato, and spinach (Mowlick et al., 2014; Liu et al., 2018; Lee et al., 2020; Zavatta et al., 2021). The benefits of RSD in suppressing soil-borne pathogens and safety have led to its widespread use in organic agricultural systems (Priyashantha and Attanayake, 2021).

The main RSD phytopathogen inhibition mechanism is the release of toxic gasses and the accumulation of organic acid during labile carbon microbial degradation under flooded conditions (Rosskopf et al., 2015). In RSD, the potential to control phytopathogens with volatile compounds is greatly influenced by the carbon source used (Shennan et al., 2017; Mahalingam et al., 2020). There is considerable evidence that organic matter is important for disease control in anaerobic environments (Liu et al., 2016; Shrestha et al., 2018; Gilardi et al., 2020; Vecchia et al., 2020). Wheat bran-based amendment to RSD at low C:N ratio (C/N) reduced *Cyperus esculentus* reproduction compared with non-amended control (Shrestha et al., 2018). Liu et al. (2016) reported that carbon source incorporated in RSD with lower C/N and higher easily oxidized carbon (EOC) may lead to greater disease control efficacy. Generally, RSD uses two kinds of carbon sources, firstly, liquid and easily degradable compounds, such as diluted ethanol (Fujita et al., 2020) and molasses (Butler et al., 2012) and secondly, solid agricultural wastes, such as livestock excrement (Zhao et al., 2021) and plant residues (Testen et al., 2021).

As a soil ecosystem disturbance, RSD offers opportunities to study how soil microbes respond to organic carbon input under anaerobic conditions (Poret-Peterson et al., 2020). In RSD-treated soils, bacterial taxa associated with *Acidobacteria*, *Firmicutes*, and *Bacteroidetes* become predominant and contribute greatly to suppressing pathogens through their physiological activities (e.g., fermentation) (Hewavitharana and Mazzola, 2016; Hewavitharana et al., 2019). Previous studies show that carbon sources with dissimilar decomposition characteristics, such as EOC content and C/N, affected soil microbial communities differently after RSD treatment (Zhao et al., 2018, 2020; Huang et al., 2019a). For example, Huang et al. (2019a) and Zhao et al. (2018) reported that plant residue-added RSD soils showed similar bacterial and fungal community structures, but differed from ethanol-added soils. Zhao et al. (2020) revealed that diversity and decomposability of organic materials added in RSD may determine the extent of microbial activity improvement. Meanwhile, soil abiotic factors, such as pH and carbon content, can also highly influence the dissimilarity and assembly of soil microbial communities in RSD-treated soil (Liu et al., 2019, 2022; Ali et al., 2022). Besides, a growing number of studies suggest that RSD-regulated soil microbial communities may deteriorate to a state similar to those of diseased soils after crop cultivation, which might be affected by root exudates (Mowlick et al., 2013; Liu et al., 2018; Huang et al., 2019a). Huang et al. (2019a) reported that bacterial and fungal diversity and community structure tended to be similar during 11 months of *Lisianthus* cultivation. Liu et al. (2018) reported that a 90-day watermelon cultivation homogenized microbial communities between RSD-treated and untreated soil, possibly because root exudates were released. Therefore, existing studies indicate that carbon source property, crop cultivation, and soil chemical property can significantly influence soil microbial communities, however their contribution rates remain unclear.

In this study, two simple-and four complex-carbon organics were chosen as the carbon source amended in RSD treatment, then two consecutive seasons of pepper pot experiments were conducted to build a RSD-cropping system. The aims were to: (i) investigate the impact of carbon source on the assembly of microbial communities in the RSD-cropping system; (ii) clarify the main contributors to the dissimilarity of soil microbial communities; and (iii) assess the key impact factor that may regulate pathogen reproduction and crop performance. We hypothesized that microbial community re-structuring in RSD-treated soil after continuous cropping maybe influenced by the comprehensive effects of carbon source property, crop cultivation, and soil chemical property.

## 2. Materials and methods

### 2.1. Soil sampling

Soil samples were collected in Lanxi County, Zhejiang Province, China (29°10′45″N, 119°16′42″E), where pepper (‘Hangjiao No.2’) has been continuously planted for many years and where some crops showed obvious *Fusarium* wilt symptoms. There was a highly abundant population of *Fusarium* in the soil (1.28 × 10^4^ colony forming units [CFU] g^−1^ dry soil). Soil chemical properties were as follows: pH 5.90, electrical conductivity (EC) 0.35 mS cm^−1^, and total organic carbon (TOC) and total nitrogen (TN) of 15.45 and 1.43 g kg^−1^, respectively.

### 2.2. Experimental design

Two simple-carbon (ethanol, glucose) and four complex-carbon (alfalfa, wheat bran, rice bran, and sugarcane residue) organics were chosen, which were widely used in RSD practice. Seven treatments were established: (1) Ck, soil was saturated with water and mulched with polyethylene (0.08 mm thickness); (2) Et, soil was saturated with 1% ethanol and mulched with polyethylene; (3–7) Gl, Al, Wh, Ri, and Su, soils were mixed with glucose, alfalfa, wheat bran, rice bran, and sugarcane residue, respectively, saturated with water, and mulched with polyethylene. The properties of the six carbon sources are described in Table 1, and the carbon amendment rate was 4 mg C g^−1^ soil for each RSD treatment. There were three replicates of each treatment, with four pots (13 × 21 cm, diameter × height) in each replicate. During the 15-day incubation period, each pot was maintained at an air temperature of 30°C to 35°C in a greenhouse. Polyethylene mulches were removed after treatment and soils were naturally drained. After RSD treatment, each pot of soil samples was collected, and the four pots of each replicate were mixed together to form one composite soil sample. The soil samples were then stored at 4°C and − 20°C for further analysis.

**Table 1:** Carbon source properties and treatment abbreviations.

Sample ID	Description	Carbon source property^a^				
		Org TOC (g kg−^1^)	Org TN (g kg−^1^)	Org C/N	Org EOC (g kg−^1^)	Diameter (cm)
Ck	No amended carbon source, flooded, and covered	/	/	/	/	/
Et	Soil flooded with 1% ethanol, and covered	406.00	0.00	/	303.30	/
Gl	Soil amended with glucose, flooded, and covered	392.35	0.00	/	243.87	/
Al	Soil amended with alfalfa, flooded, and covered	368.57	22.74	16.21	89.70	<0.2
Wh	Soil amended with wheat bran, flooded, and covered	363.93	11.05	32.93	99.23	<0.2
Ri	Soil amended with rice bran, flooded, and covered	351.02	4.15	84.66	43.17	<0.2
Su	Soil amended with sugarcane residue, flooded, and covered	389.85	3.28	118.85	74.56	<0.2

^a^Org TOC, Org TN, Org C/N, and Org EOC represent total organic carbon, total nitrogen, C/N, and easily oxidized carbon of carbon source, respectively.

A pepper seedling (‘Hangjiao No.2’) was planted in each pot and cultivated for 3 months from August 2020 to November 2020. The experiment was entirely randomized, and all pots were grown in a greenhouse at 28°C during the day and 20°C at night. During the cultivation period, the soils were regularly watered to maintain a suitable moisture level. At the time of flowering, each pot was fertilized with an inorganic compound fertilizer (3 mg, N: P: K = 16:16:16) every 7 days. Crop performance indices (stem diameter, plant height, aboveground biomass, and crop yield) were measured after planting. Later, the soil in each pot was thoroughly mixed and pepper seedlings were planted again in the same soil from April 2021 to July 2021. We collected and stored rhizosphere soil samples as described above.

### 2.3. Soil chemical property detection

Measurements were taken at a ratio of 1:2.5 (m/v) of soil to water to determine pH and EC using a PB-10 pH meter (Sartorius AG, Goettingen, Germany) and DDSJ-308F conductivity meter (Measuretech, Shanghai, China), respectively. Soil TOC was measured using the potassium dichromate volumetric method (Kolthoff and Lingane, 1933). EOC of carbon sorce (Org EOC) was determined with 333 mmol L^−1^ of KMnO_4_ using a spectrophotometer according to Liu et al. (2016). Soil TN and TN of carbon sorce (Org TN) were determined using Kjeldahl-N method (Sader et al., 2004). Utilizing the Kjeldahl-N method, molybdenum-antimony anti-colorimetry, and flame photometry, soil available nitrogen (AN), available phosphorus (AP), and available potassium (AK), respectively, were determined (Bao, 2008).

### 2.4. Number of cultivable *Fusarium*

The number of cultivable *Fusarium* was determined using a *Fusarium*-selective medium described in our previous research (Zhu et al., 2022). Five grams of soil collected at the end of anaerobic treatment and crop cultivation stages was re-suspended in 45 ml 0.85% NaCl and shaken continuously for 30 min in a sterile Erlenmeyer flask. Three duplicates of each sample were plated onto *Fusarium*-selective media after serial dilution with sterile saline. Streptomycin sulfate (0.75 μg ml^−1^) was added to each medium to inhibit bacterial growth. Plates were incubated for 4 days at 30°C, and colony forming units (CFU) per gram of dry soil were then counted.

### 2.5. DNA extraction and PCR amplification

Genomic DNA was isolated from 0.5 g soil taken after anaerobic treatment and after two planting seasons using a Power Soil™ DNA Isolation Kit (MO BIO Laboratories, Carlsbad, CA, United States). Isolated DNA was determined using a UV–Vis spectrophotometer from NanoDrop Technologies (Wilmington, DE, United States), and then used as a template for further sequencing. For bacteria, primer sets 338F (5’-ACTCCTACGGGAGGCAGCA-3′) and 806R (5’-GGACTACHVGGGTWTCTAAT-3′) were used to amplify the V3–V4 hypervariable regions (Zhang et al., 2017). For fungi, the ITS1 region was amplified using primers ITS5F (5’-GGAAGTAAAAGTCGTAACAAGG-3′) and ITS1R (5’-GCTGCGTTCTTCATCGATGC-3′) (Hou et al., 2021). PCR reaction mixture and thermal profiles were performed according to Zhu et al. (2022). PCR product amplifications were followed by purification, quantification, and mixing to achieve equal concentrations.

### 2.6. Sequencing and data processing

The mixtures were sequenced in a paired end format using the Illumina MiSeq platform by Personalbio Technology Co. Ltd. (Shanghai, China). Sequencing data analyzes were performed using the Quantitative Insights into Microbial Ecology 2 (QIIME2, version 2019.4) platform (Bolyen et al., 2019). Firstly, fastq sequence files with paired-end primers were trimmed using the “qiime cutadapt trim-paired” command. After that, reads were de-noised in DADA2 (Callahan et al., 2016) pipeline using the “qiime dada2 denoise-paired” command, then each amplicon sequence variant (ASV) was represented by a sequence and a feature table was created. Using the “qiime feature-table summarize” command, a summary report listing the sequences associated with each sample was generated following the DADA2 denoising step. To generate a rarefied feature table, all samples were rarefied into the same depth using the “qiime feature-table rarefy” command. Alpha and beta diversity were calculated from the rarefied feature table using the “qiime diversity alpha” and “qiime diversity beta” commands. Taxonomy was assigned to ASVs using the “qiime feature-classifier classify-sklearn” command against the SILVA release 132 (Quast et al., 2012) and UNITE release 8.0 (Kõljalg et al., 2013) databases. The sequencing data of 16 s rRNA and ITS rRNA genes are available at the NCBI Sequence Read Archive (SRA) database under accession number PRJNA882600 and PRJNA882628, respectively.

### 2.7. Data analysis

Significant differences in soil chemical properties, microbial characteristics and crop performance indices among treatments were analyzed with one-way ANOVA using Turkey’s HSD in SPSS 20.0 (SPSS Inc., Chicago, IL, United States). Analysis of similarities (ANOSIM) among treatments and non-parametric multivariate analysis of variance (PERMANOVA) were performed to assess statistical significance using the *vegan* package in R (Oksanen et al., 2016). Variation partition analysis (VPA) was performed with the *vegan* package in R using the varpart function to determine how carbon source, crop cultivation, and soil chemical property affected bacterial and fungal community structures. Also, Mantel tests were conducted using the mantel function in R to assess Pearson’s rank correlations between bacterial and fungal communities and environmental dissimilarity matrices. Moreover, we explored the relationships between bacterial and fungal community compositions and impact factors through redundancy analysis (RDA) using the *vegan* package in R. Pearson correlation coefficients among carbon source property, soil chemical property, microbe and crop yield were conducted in R.

## 3. Results

### 3.1. Soil chemical properties

**Figure 1:**
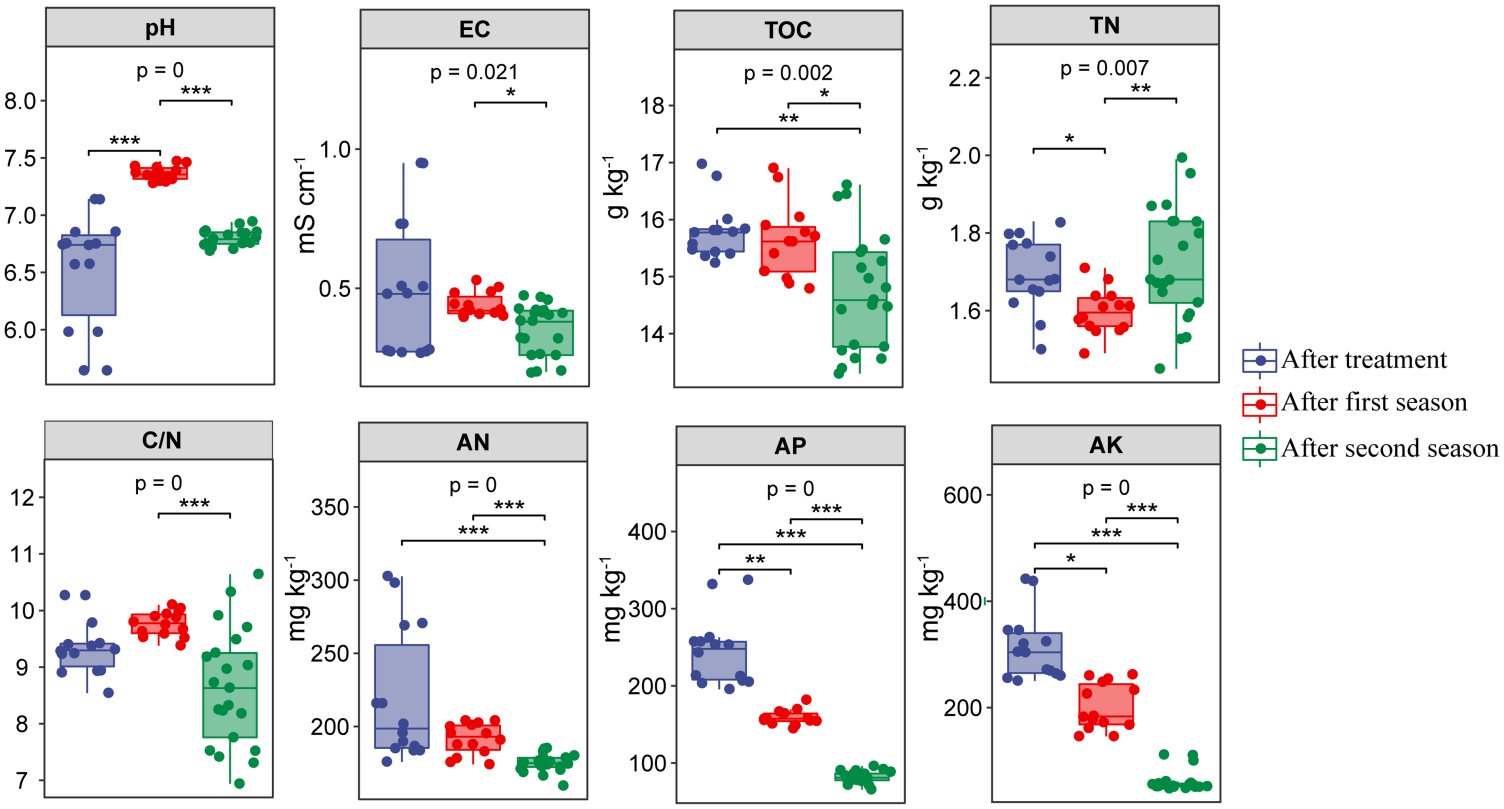
Chemical properties in different soils after RSD treatment and two planting seasons. EC: electric conductivity, TOC: exchangeable calcium, TN: total nitrogen, C/N: carbon to nitrogen ratio, AK: available nitrogen, AP: available phosphate, AK: available potassium. The significance of different groups was determined by the *p* values (ANOVA, Tukey’s HSD). ^*^p < 0.05; ^**^p < 0.01; ^***^p < 0.001.

Chemical properties in different soils after RSD treatment and two planting seasons are shown in Supplementary Table S1. Soil chemical properties varied significantly in RSD-treated soils amended with different carbon sources. After RSD treatment, soil TOC, TN, AN, and AK were significantly (*p* < 0.05) higher in Al than in the other soils. In addition, pH in complex-carbon soils (Al, Wh, Ri and Su) was significantly (*p* < 0.05) higher than in simple-carbon soils (Et and Gl), while EC showed the opposite trend. After crop cultivation, most of the chemical indices in different treatments also varied significantly (*p* < 0.05), except soil C/N and AP in the first season and soil AN and AP in the second season. Noticeably, TOC in the complex-carbon soils was significantly (*p* < 0.05) higher than in the simple-carbon soils after two planting seasons. Overall, soil pH significantly (*p* < 0.001) increased, and soil TN, AP, and AK significantly (*p* < 0.05 or *p* < 0.01) decreased at the end of the first season compared with those at the end of RSD treatment (Figure 1). After two seasons, soil TOC, AN, AP, and AK significantly (*p* < 0.05) decreased, while soil pH, TN, and C/N returned to initial levels, compared with those detected at the end of RSD treatment.

### 3.2. Soil microbial community characteristics

#### 3.2.1. MiSeq sequencing data

**Figure 2:**
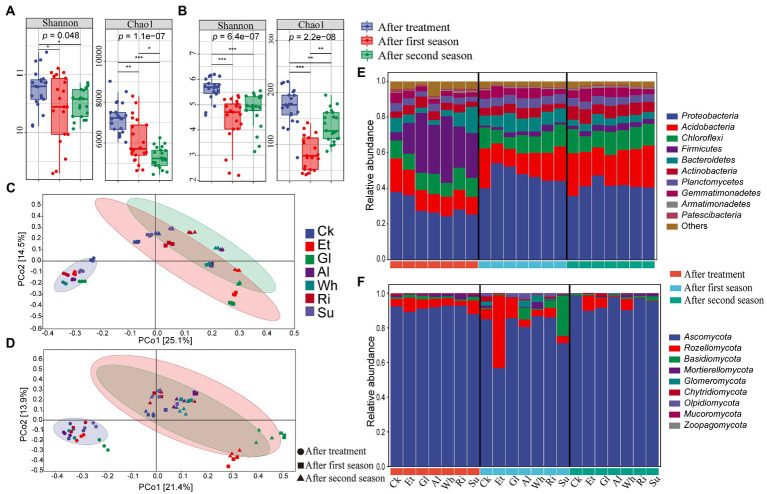
**(A,B)** Alpha diversity indices of bacterial and fungal communities in different soils collected after RSD treatment and two planting seasons. The significance of different groups was determined by the *p* values (ANOVA, Tukey’s HSD). ^*^p < 0.05; ^**^p < 0.01; ^***^p < 0.001. **(C,D)** Principal coordinates analyzes (PCoA) were conducted based on soil bacterial and fungal ASVs using Bray-Curtis distance. **(E,F)** Soil bacterial and fungal community compositions at phylum level in different soils collected after RSD treatment and two planting seasons. Treatment abbreviations are defined in Table 1.

Totally, 5,322,386 and 3,560,817 high-quality sequences of the 16S and ITS genes, respectively, were obtained from the 63 soils (7 treatments × 3 biological replicates × 3 sampling time). After rarefying, 323,817 bacterial and 6,804 fungal ASVs were obtained (Supplementary Table S2). All samples had coverage levels above 95.0%, indicating sufficient sequencing depth. After the first season, Shannon and Chao1 indices of both bacteria and fungi significantly (*p* < 0.05) decreased compared with those at the end of RSD treatment (Figures 2A,B). After the second season, the bacterial Chao1 index continued to decrease markedly (*p* < 0.001), while Shannon and Chao1 indices of fungi recovered somewhat, although still significantly (*p* < 0.05) lower than at the end of RSD treatment. Noticeably, Shannon and Chao1 indices of both bacteria and fungi in Et were lowest after the first and second seasons (Supplementary Table S3).

Principal coordinate analysis (PCoA) showed that bacterial and fungal communities were significantly different among soils collected after RSD treatment and two planting seasons, which was confirmed by two complementary nonparametric multivariate statistical tests (*p* < 0.01 by Adonis and ANOSIM; Figures 2C,D; Supplementary Table S4). Simultaneously, the impact of different carbon sources on soil microbial community dissimilarity after RSD treatment and the subsequent cultivation process were also analyzed. After RSD treatment, soil bacterial and fungal communities in simple-carbon soils were significantly different (*p* < 0.01) to those in complex-carbon soils. After crop cultivation, soil bacterial and fungal communities between simple-and complex-carbon soils differed significantly along PC axes as confirmed by Adonis and ANOSIM results (*p* < 0.01 for bacteria and fungi in both first and second season).

#### 3.2.2. Soil microbial community compositions

For the bacterial ASVs, *Proteobacteria*, *Acidobacteria*, *Chloroflexi*, *Firmicutes*, and *Bacteroidetes* were the five most abundant bacteria phyla, accounting for 76.19% ~ 85.67% of the total sequences (Figure 2E). Specifically, *Proteobacteria* and *Firmicutes* were two dominant bacterial phyla in soils after RSD treatment, while *Acidobacteria* replaced *Firmicutes* as the dominant bacterial community after crop cultivation. For fungi, 95.19% ~ 99.94% of the total sequences were classified as members of the *Ascomycota*, *Rozellomycota*, *Basidiomycota*, *Mortierellomycota*, and *Glomeromycota* (Figure 2F). *Ascomycota* was the dominant fungal phylum across all samples, and accounted for 56.74 to 98.55% of all fungal sequences. Remarkably, *Rozellomycota* and *Basidiomycota* dominated in Et and Su at the first season, respectively. Dominant relative abundance (RA) of both bacterial and fungal genera in the different soils also varied remarkably after RSD treatment and crop cultivation (Supplementary Figure S1). For example, RA of bacterial genus *Azotobacter* and fungal genus UC_*Sordariaceae* dominated in Et at the first and second seasons, while RA of fungal genus *Zopfiella* dominated in Ri and Su.

### 3.3. Factors influencing soil bacterial and fungal communities

**Figure 3:**
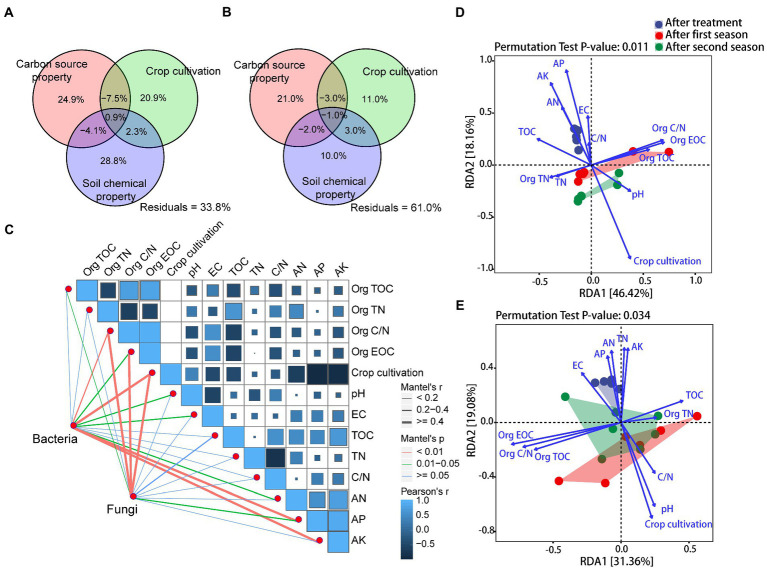
**(A,B)** Variance partitioning analysis (VPA) of the effects of carbon source property, crop cultivation, soil chemical property, and their interactions on soil bacterial and fungal communities, **(C)** Pearson’s correlation coefficients of bacterial and fungal ASVs and impact factors based on Mantel tests, and **(D,E)** Redundancy analysis (RDA) of bacterial and fungal ASVs and impact factors. Impact factor abbreviations are defined in Table 1 and Figure 1.

Variation partitioning analysis (VPA) revealed that carbon source property, crop cultivation, and soil chemical property explained 66.2 and 39.0% of the observed variation in the bacterial and fungal communities, respectively (Figures 3A,B). Soil chemical property was the primary dominant influence on bacterial community (28.8% of the variation, *p* = 0.001 based on the Mantel test, Supplementary Table S5), while carbon source property and crop cultivation also significantly influenced it (24.9 and 20.9% of the variation, *p* = 0.045 and *p* = 0.001, respectively). However, fungal community was greatly influenced by carbon source property and crop cultivation (21.0 and 11.0% of the variation, *p* = 0.001 and *p* = 0.001, respectively), while soil chemical property exhibited no significant impacts on it (10.0% of the variation, *p* = 0.097).

To explore the major impacts on bacterial and fungal community assemblages, we also conducted Mantel tests comparing the bacterial and fungal community compositions (based on Bray-Curtis distance matrices) with 13 variables (based on Euclidean distance matrices). As shown in Figure 3C and Supplementary Table S6, Org C/N (*r* = 0.250, *p* = 0.006), crop cultivation (*r* = 0.721, *p* = 0.001), soil AP (*r* = 0.555, *p* = 0.001), and soil AK (*r* = 0.497, *p* = 0.001) were the most important factors shaping bacterial community composition, while Org C/N (*r* = 0.401, *p* = 0.002), Org EOC (*r* = 0.424, *p* = 0.001), and crop cultivation (*r* = 0.405, *p* = 0.001) were the most important factors shaping fungal community composition. When subject to RDA models, the 13 detected factors explained ~65% of bacterial community variation and ~ 52% of fungal community variation (Figures 3D,E). Specifically, soil bacterial community variation was mainly driven by Org C/N, Org EOC, crop cultivation, soil AP, and soil AK, while Org TOC, Org C/N, Org EOC, crop cultivation, and soil pH were significant drivers of fungal community changes (*p* < 0.05; Supplementary Table S7).

### 3.4. *Fusarium* reproduction and crop performance

**Figure 4:**
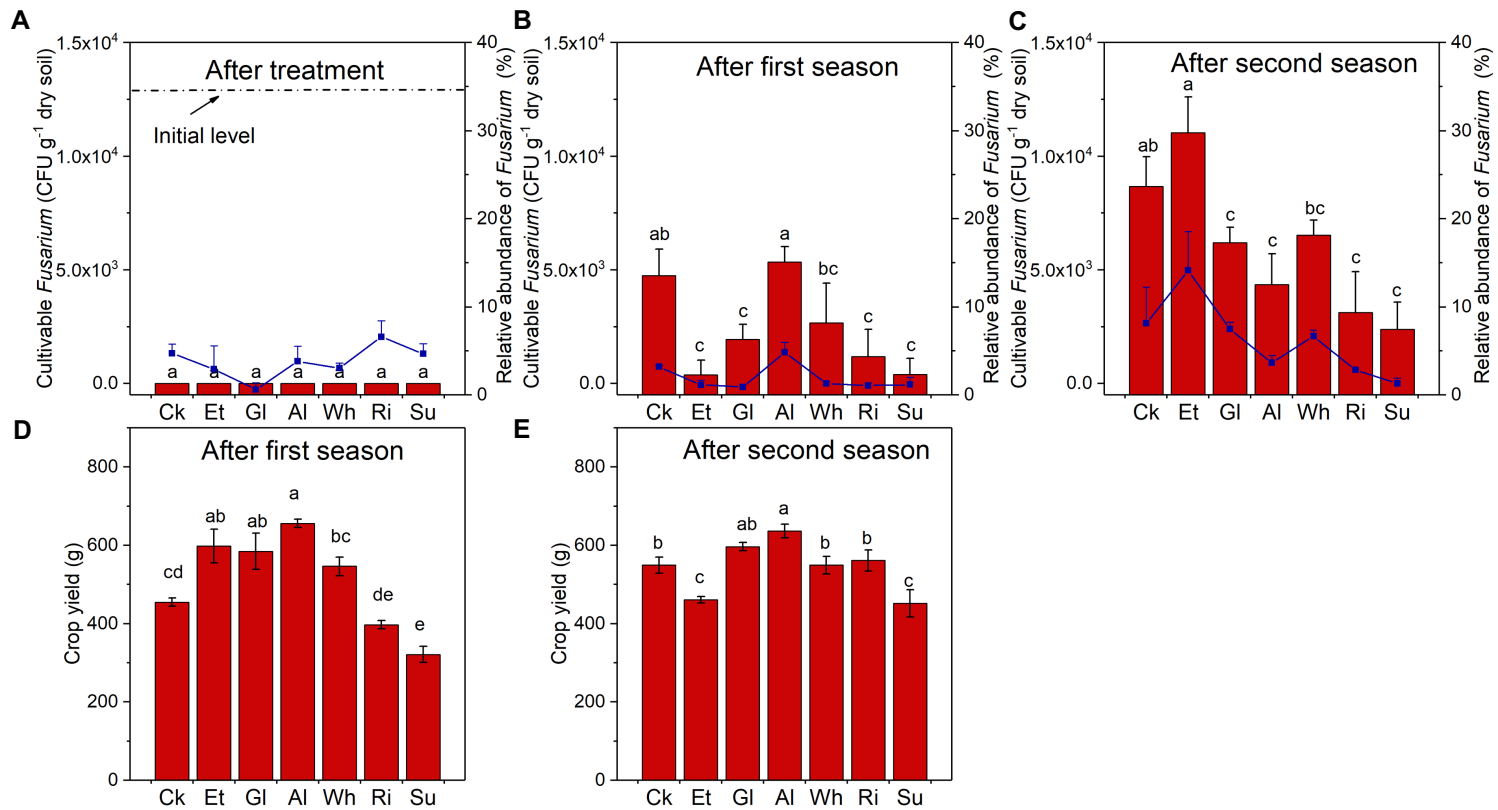
**(A–C)** The number of cultivable *Fusarium* and relative abundance of *Fusarium* in soils collected after RSD treatment and two planting seasons. **(D,E)** Crop yield after the first and second seasons. Different letters indicate significant differences among the soils based on Tukey’s HSD (*p* < 0.05). Error bars indicate SDs. Treatment abbreviations are defined in Table 1.

The changes of *Fusarium* abundance and crop yield in different treatments during RSD treatment and two planting seasons were also investigated. During the 15-day RSD treatment period, the number of cultivable *Fusarium* in most soils (except for Ck) decreased to 0 at the 5th day (Supplementary Figure S2). After RSD treatment, no cultivable *Fusarium* was detected in any of the soils, while RA of *Fusarium* was highest in Ri and negatively correlated with Org EOC (Figure 4A; Supplementary Table S8). In addition, the number of cultivable *Fusarium* after the first season was highest in Al and after the second season, highest in Et, while RA of *Fusarium* showed the same trend (Figures 4B,C). As for crop yield, it was higher in the Et, Gl, and Al after the first season, and it was highest in Al and lower in Et and Su after the second season (Figures 4D,E). The crop yield of complex-carbon soils after the first season was positively affected by Org TN and negatively affected by Org C/N (Supplementary Table S9).

**Figure 5:**
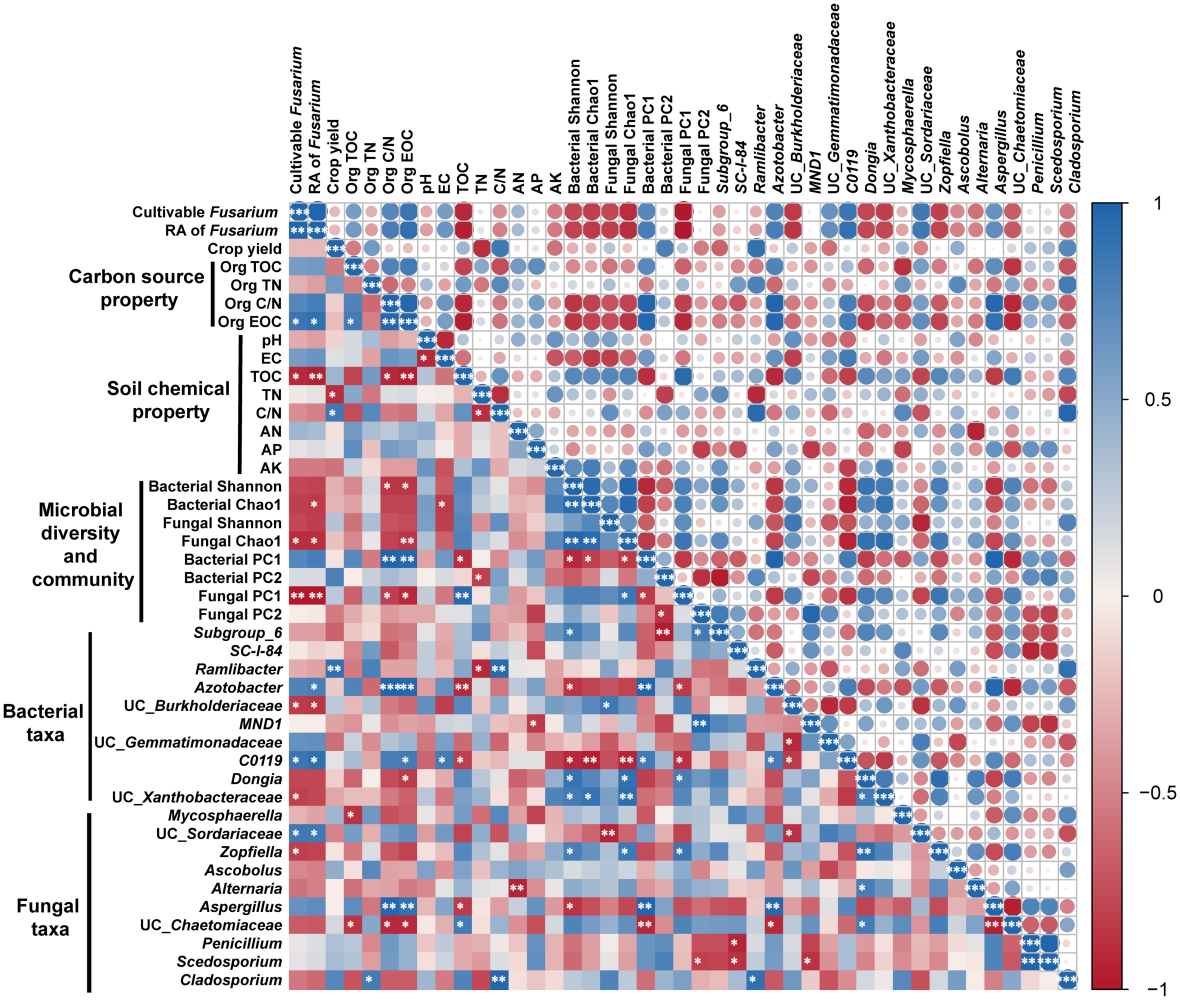
Relationships among microbe, crop yield, carbon source property, and soil chemical property after two planting seasons. Circle color and size represents Pearson correlation coefficients (*r*). ^*^p < 0.05; ^**^p < 0.01; ^***^p < 0.001. Only the top 10 bacterial and fungal genera are shown. Impact factor abbreviations are defined in Table 1 and Figure 1.

After two consecutive seasons of planting, relationships among microbe, crop yield, carbon source property, and soil chemical property were further explored (Figure 5). The results showed that the number of cultivable *Fusarium* was positively and significantly correlated with RA of *Fusarium*. Moreover, the number of cultivable *Fusarium* was positively correlated with Org EOC, and negatively correlated with soil TOC, Fungal Chao1, Fungal PC1, the bacterial genera UC_*Burkholderiaceae* and UC_*Xanthobacteraceae*, and the fungal genus *Zopfiella*. Overall, crop yield was negatively affected by soil TN and positively affected by soil C/N, while no significant relationship was found between crop yield and carbon source property.

## 4. Discussion

### 4.1. The effects of carbon source property, crop cultivation, and chemical property on soil bacterial and fungal communities

Numerous studies have reported that soil microbiomes are remarkably altered by RSD (Zhou et al., 2019; Lee et al., 2020; Poret-Peterson et al., 2020). However, knowledge of soil microbial community assemblages in RSD-treated soils amended with different carbon sources after continuous crop cultivation is still limited. In this study, VPA showed that bacterial community was significantly affected by carbon source, crop cultivation, and soil chemical property. Nevertheless, fungal community structure was only significantly related to carbon source and crop cultivation. Soil chemical property, explaining 28.8% of bacterial community variation, played a very important role on bacterial community, while no significant influence was found on the fungal community. This is consistent with previous studies revealing that bacterial community assembly is more dependent on soil abiotic characteristics than fungal community assembly (Liu et al., 2019, 2022). In addition, we found soil nutrient indices, such as AP and AK, were the most important factors in describing soil bacterial community patterns. Besides, crop cultivation could also significantly affect bacterial and fungal community structures in the RSD-cropping system. This might be attributed to the crucial role of root exudates in regulating microbe colonization of the rhizosphere as well as activating native microbes (Wang et al., 2021).

More importantly, the reestablishment of bacterial and fungal communities, were significantly affected by carbon source property in the RSD-cropping system. The influence of carbon source property on soil microorganism assemblages may be attributed to two aspects: Firstly, carbon source has direct effects on soil microbial community structures. PCoA revealed that soil microbial communities between the complex-carbon and simple-carbon samples were significantly different after RSD treatment and crop cultivation, and the results of Mantel tests confirmed that Org C/N and Org EOC were significant factors affecting soil bacterial and fungal communities. It seems that microbial decomposition of different carbon sources produces different intermediate metabolites, thus shaping dissimilar microbial communities (Liu et al., 2016). Secondly, different carbon source properties can significantly influence soil chemical properties, then indirectly affect soil microorganism assemblages (Li et al., 2018; Schlatter et al., 2022). As demonstrated in Supplementary Figure S3, bacterial or fungal community structure in soil collected after RSD treatment, first season, and second season, respectively, was significantly correlated with soil EC, AK, and TOC, and these chemical indices were significantly affected by carbon source Org EOC and Org C/N. Overall, this study highlights the important roles of carbon source property, crop cultivation, and soil chemical property on soil microbial community re-structuring in the RSD-cropping system.

### 4.2. *Fusarium* changes in different soils after RSD treatment and crop cultivation

There is increasing evidence that RSD practice incorporated with organic substances often effectively reduces the pathogen population (Momma et al., 2013; Huang et al., 2015, 2016). In this study, RSD effectively killed cultivable *Fusarium* in all soils amended with different carbon sources. Nevertheless, RA of *Fusarium* was quite different, and it was negatively correlated with Org EOC. For instance, RA of *Fusarium* was lower in complex-carbon soils (Et and Gl), and higher in complex-carbon soils (Ri and Su). According to the findings of Liu et al. (2016) and Huang et al. (2016), carbon source with higher Org EOC could lead to more toxic organic acid production and, therefore, result in higher sterilization efficiency during the RSD process. In the present study, pH values in simple-carbon soils (Et and Gl) were lower than in complex-carbon soils (Al, Wh, Ri, and Su) during the 15-day RSD treatment (Supplementary Figure S4), possibly because the former produced more organic acids (Huang et al., 2016; Liu et al., 2016). Correspondingly, highly recalcitrant substrates do not yield a significant amount of anaerobic decomposition metabolites due to their slow decomposition rates (Shrestha et al., 2021).

In addition, previous studies have reported that soil-borne pathogens can be restored with plant cultivation (Zhao et al., 2017; Liu et al., 2018). After two planting seasons, the number of cultivable *Fusarium* in most of the soils (Gl, Al, Ri, and Su) was still maintained at a lower level, while that in Et was the highest. Correlation analysis showed that the number of cultivable *Fusarium* was positively correlated with Org EOC, and negatively correlated with soil TOC (Dignam et al., 2018; Cao et al., 2022). This finding suggests that heuristically, the higher Org EOC of carbon source involved in RSD treatment, the more likely pathogen reproduction occurs during the subsequent crop cultivation. According to previous research, complex-carbon is comprised of lignin, cellulose, and hemicellulose, and therefore much more difficult to decompose and utilize than simple-carbon, thereby exhibiting persistent inhibition of pathogen reproduction (Huang et al., 2019b). Meanwhile, the number of cultivable *Fusarium* was also negatively correlated with Fungal Chao1 and Fungal PC1. As commonly accepted, species richness may promote antibiotic production as well as community-wide antagonistic interactions, which can help suppress pathogens (Saleem et al., 2019). Thus, the lowest level of fungal diversity and richness in Et after two consecutive seasons of planting, suggests possible mechanisms by which microbial diversity loss may directly affect pathogen selection and persistence (Flanagan et al., 2007). Although earlier studies confirmed the role of Org EOC in antifungal efficiency at the RSD treatment stage, our study found that carbon source with relatively lower Org EOC is more conducive to pathogen reproduction inhibition during the subsequent crop cultivation process. Furthermore, we demonstrated for the first time, the links between fungal community richness and pathogen reproduction, which further highlights the vital role of fungi in the RSD-cropping system amended with different carbon sources.

### 4.3. The impact of carbon source property on crop performance

It is well known that RSD can ameliorate barrier soil physiochemical properties (Di Gioia et al., 2017; Meng et al., 2022), however, crop performance after consecutive crop cultivation in RSD-treated soils incorporated with different carbon sources remains poorly understood. In this study, we found that crop yield of pepper plants was positively affected by Org TN and negatively affected by Org C/N among the complex-carbon soils (Al, Wh, Ri, and Su) after the first season. Similarly, Shrestha et al. (2021) also revealed that C/N of carbon source (Org C/N) amended in RSD exerts a strong influence on soil inorganic N and crop performance. After the first season, crop yield was highest in Al, and lower in Ri and Su. According to Spohn (2015) and Sinsabaugh et al. (2013), C/N of organic substrate above 20 results in microbial N limitations on decomposition and reduces soil inorganic N availability. Alfalfa, with the lowest Org C/N (16.21), could favor biological decomposition and improve microbial activity when amended in soil for RSD, then mineralize sufficient N to satisfy plant growth demands (Luo et al., 2018; Mao et al., 2022). Correspondingly, the highest TN and AN content were found in Al after RSD treatment, with the highest crop yield after cultivation. On the contrary, soil TN and AN content were lower in Ri (Org C/N = 84.66) and Su (Org C/N = 118.85) after RSD treatment, and the crop yield of the first season in Ri and Su also showed lower amount than in other soils. However, no significant correlation was found between crop yield and carbon source property in the second season, indicating that the direct effect of carbon source property on crop yield only lasted for one season. The results of correlation analysis revealed that the crop yield at the second season was significant affected by soil TN and C/N, and it was highest in Al, and lower in Su. Moreover, lower crop yield was also found in Et after two consecutive seasons of planting. On one hand, soil TOC was lowest in Et with the highest Org EOC (303.30). As soil TOC is a key factor in nutrient storage and cycling, plenty of research have demonstrated the important role of organic C in maintaining crop yeild (Pan et al., 2009; Oldfield et al., 2019; Kopittke et al., 2022). Compared to the simple-carbon, complex-carbon with lower decomposition efficiency usually has a retention effect in soil (Liu et al., 2016), and the result in Figure 5 provides direct evidence that soil TOC was negatively correlated with Org C/N and Org EOC. On the other hand, soil TOC decrease adversely affected fungal community assemblages, such as the sharp decrease of Fungal Shannon and Chao1 indices, which further aggravated *Fusarium* reproduction (Saleem et al., 2019). Finally, the combination of these factors may lead to the lower crop yield in Et. Although no direct correlation was detected between carbon source property and crop yield after consecutive cropping, Org C/N and Org EOC of carbon source could regulate soil chemical and biological properties, then play important roles in crop performance.

## 5. Conclusion

**Figure 6:**
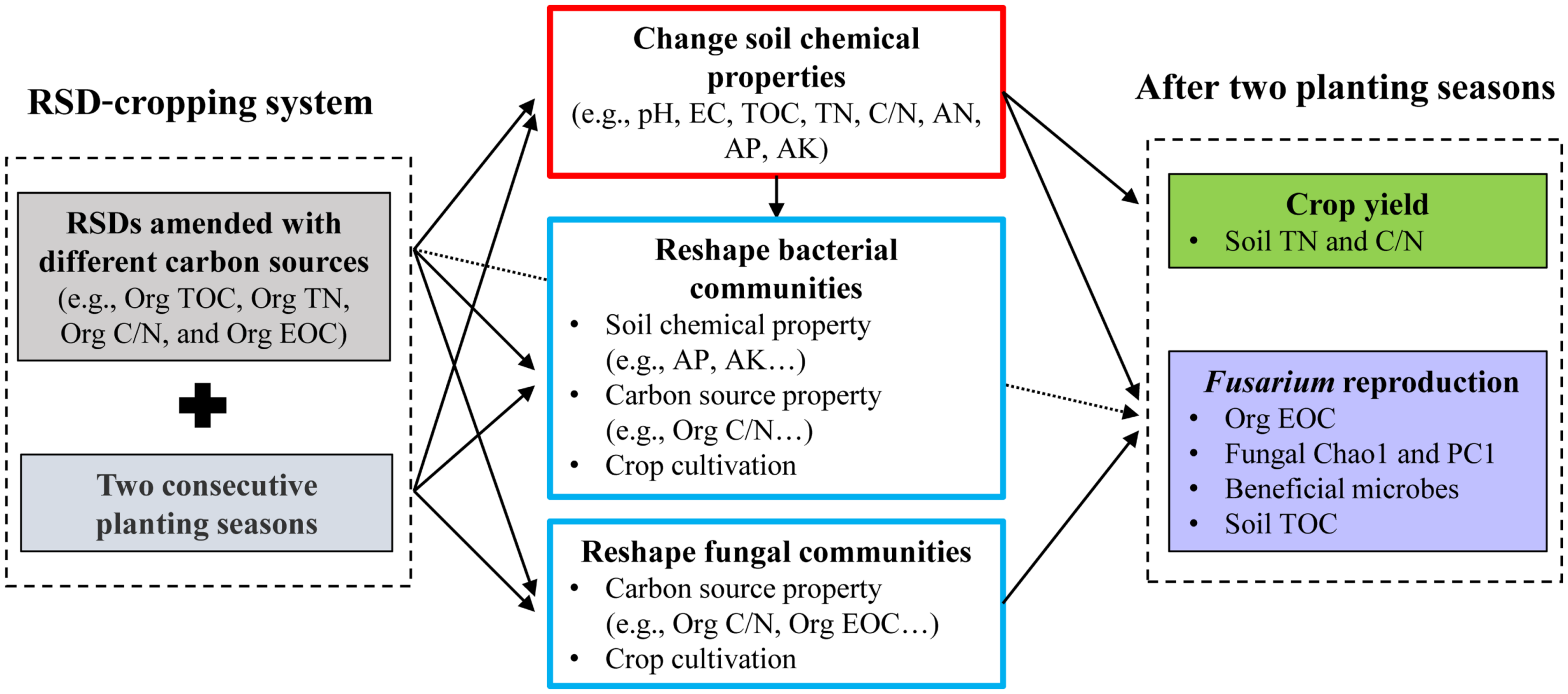
Conceptual diagram describing the influence of carbon source on soil microbial community reestablishment, soil chemical properties, pathogen reproduction, and crop performance in a RSD-cropping system.

A conceptual diagram (Figure 6) was constructed to show the influence of carbon source on soil microbial community reestablishment, soil chemical properties, pathogen reproduction, and crop performance in a RSD-cropping system. Bacterial community structure was significantly affected by soil chemical property, carbon source property, and crop cultivation, while fungal community structure was only influenced by carbon source property and crop cultivation. Carbon source properties, such as Org C/N and Org EOC, were the most import factors affecting the distribution of bacterial and fungal communities. After two planting seasons, *Fusarium* reproduction was positively correlated with Org EOC, and negatively correlated with soil TOC, fungal Chao1 and fungal PC1. Crop yield was highest in Al, and lower in Et and Su after consecutive cropping, and it was significantly correlated with soil TN and C/N. Comprehensively, Org C/N and Org EOC of carbon source incorporated in RSD, are very important for soil microbe reestablishment, pathogen reproduction, and crop performance. Future studies should focus on the effects of carbon source properties on soil functional core microbiome during RSD process, and link them to soil disease resistance and growth promotion.

## Data availability statement

The datasets presented in this study can be found in online repositories. The names of the repository/repositories and accession number(s) can be found in the article/Supplementary material.

## Author contributions

WZ and YY conceived and designed the research. WZ, XL, and CH conducted experiments. FZ and LH performed statistical analyzes. WZ wrote the first draft of the manuscript. SZ and YY wrote sections of the manuscript. All authors have read and approved the final manuscript.

## Funding

This work was financially supported by Natural Science Foundation of Zhejiang Province (LQ21D010002), Science and Technology Program of Zhejiang Province (2020C02030), Cooperative Extension Project of Major Agricultural Technologies of Zhejiang Province (2019XTTGSC04-3), Ningbo Public Welfare Science and Technology Plan Project (2021S023), and Department of Agriculture and Rural Development of Zhejiang Province (2022SNJF024).

## Conflict of interest

The authors declare that the research was conducted in the absence of any commercial or financial relationships that could be construed as a potential conflict of interest.

## Publisher’s note

All claims expressed in this article are solely those of the authors and do not necessarily represent those of their affiliated organizations, or those of the publisher, the editors and the reviewers. Any product that may be evaluated in this article, or claim that may be made by its manufacturer, is not guaranteed or endorsed by the publisher.
