# Supplementary material for: Contributions of carbon source, crop cultivation, and chemical property on microbial community assemblage in soil subjected to reductive disinfestation
Source: Front Microbiol. 2023 Feb 24;14:1146207. doi: 10.3389/fmicb.2023.1146207 (PMC10081160; doi:10.3389/fmicb.2023.1146207)
Supplement: Supplementary file 1 [file Data_Sheet_1.docx]

***Supplemental material***

**Contributions of carbon source, crop cultivation, and chemical property on microbial community assemblage in soil subjected to** **reductive disinfestation**

**Weijing Zhu^1^, Xiaolin Lu^1^, Chunlai Hong^1^, Leidong Hong^1^, Fengxiang Zhu^1^, Shuo Zhang^2^, Yanlai Yao^1*^**

^1^State Key Laboratory for Managing Biotic and Chemical Threats to the Quality and Safety of Agro-products, Institute of Environment, Resource, Soil and Fertilizer, Zhejiang Academy of Agricultural Sciences, Hangzhou 310021, China

^2^Ningbo Agricultural and Rural Green Development Center, Ningbo 315099, China

***Correspondence:**

Yanlai Yao

[yaoyl@zaas.ac.cn](mailto:yaoyl@zaas.ac.cn)

**Supplementary Table S1** Soil chemical properties after RSD treatment and two planting seasons.

|  | **Sample** | **pH** | **EC (mS/cm)** | **TOC (g/kg)** | **TN (g/kg)** | **C/N** | **AN (mg/kg)** | **AP (mg/kg)** | **AK (mg/kg)** |
| --- | --- | --- | --- | --- | --- | --- | --- | --- | --- |
| **RSD treatment** | Ck | 6.57 ± 0.02 d | 0.28 ± 0.01 e | 15.62 ± 0.54 b | 1.6 ± 0.06 c | 9.76 ± 0.73 a | 183.49 ± 0 d | 334.3 ± 3.98 a | 261.92 ± 2.89 ef |
|  | Et | 5.98 ± 0.01 e | 0.73 ± 0 b | 15.8 ± 0.04 b | 1.77 ± 0 ab | 8.92 ± 0.02 ab | 215.71 ± 0 c | 207.8 ± 6.51 c | 270.24 ± 1.45 e |
|  | Gl | 5.64 ± 0 f | 0.95 ± 0.01 a | 15.45 ± 0.03 b | 1.66 ± 0.02 bc | 9.3 ± 0.1 ab | 198.6 ± 4.32 d | 200.99 ± 7.77 c | 253 ± 3.61 f |
|  | Al | 6.85 ± 0 b | 0.48 ± 0.02 d | 16.87 ± 0.15 a | 1.81 ± 0.04 a | 9.29 ± 0.02 ab | 300.32 ± 3.24 a | 209.13 ± 5.78 c | 439.73 ± 2.89 a |
|  | Wh | 7.14 ± 0.01 a | 0.51 ± 0 c | 15.48 ± 0.12 b | 1.77 ± 0.02 ab | 8.74 ± 0.28 b | 269.67 ± 1.08 b | 257.11 ± 0 b | 345.55 ± 0 b |
|  | Ri | 6.75 ± 0 c | 0.27 ± 0 e | 15.57 ± 0.3 b | 1.59 ± 0.13 c | 9.83 ± 0.61 a | 185.72 ± 1.08 d | 258 ± 6.87 b | 322.18 ± 2.89 c |
|  | Su | 6.74 ± 0 c | 0.27 ± 0.01 e | 15.81 ± 0 b | 1.65 ± 0.04 bc | 9.6 ± 0.26 ab | 182.64 ± 9.73 d | 248 ± 7.23 b | 304.25 ± 0.72 d |
| **First season** | Ck | 7.35 ± 0.01 cd | 0.41 ± 0 c | 14.99 ± 0.15 b | 1.57 ± 0.02 bc | 9.56 ± 0.04 a | 180.53 ± 3.23 b | 154.44 ± 5.05 a | 177.38 ± 7.21 c |
|  | Et | 7.47 ± 0.01 a | 0.41 ± 0.01 c | 15.2 ± 0.58 b | 1.55 ± 0 bc | 9.78 ± 0.37 a | 193.86 ± 4.32 ab | 157.49 ± 0.58 a | 146.05 ± 0 d |
|  | Gl | 7.4 ± 0.05 ab | 0.51 ± 0.03 a | 15.03 ± 0.09 b | 1.53 ± 0.04 c | 9.86 ± 0.33 a | 182.43 ± 9.72 b | 161.9 ± 10.83 a | 164.46 ± 4.33 c |
|  | Al | 7.36 ± 0.01 cd | 0.49 ± 0 ab | 16.82 ± 0.11 a | 1.69 ± 0.02 a | 9.94 ± 0.06 a | 201.43 ± 2.16 a | 159.8 ± 6.21 a | 251.18 ± 4.33 a |
|  | Wh | 7.31 ± 0.01 c | 0.41 ± 0 c | 15.7 ± 0.11 b | 1.6 ± 0.02 abc | 9.84 ± 0.06 a | 203.97 ± 0 a | 146.66 ± 2.88 a | 180.78 ± 5.04 c |
|  | Ri | 7.29 ± 0.01 c | 0.44 ± 0 bc | 15.55 ± 0.22 b | 1.63 ± 0.02 ab | 9.57 ± 0.26 a | 201.67 ± 1.08 a | 172.92 ± 12.39 a | 229.65 ± 5.04 b |
|  | Su | 7.41 ± 0.02 ab | 0.4 ± 0 c | 15.97 ± 0.1 ab | 1.63 ± 0.02 ab | 9.80 ± 0.19 a | 198.98 ± 5.39 ab | 160.59 ± 8.37 a | 261.18 ± 1.44 a |
| **Second season** | Ck | 6.75 ± 0.01 cd | 0.41 ± 0.01 b | 13.59 ± 0.25 bc | 1.76 ± 0.08 ab | 7.79 ± 0.46 cd | 176.44 ± 3.18 a | 84.92 ± 3.02 a | 51.03 ± 0 b |
|  | Et | 6.75 ± 0.01 cd | 0.47 ± 0.01 a | 13.51 ± 0.1 c | 1.89 ± 0.06 a | 7.22 ± 0.25 d | 178.07 ± 6.88 a | 81.3 ± 13.9 a | 53.79 ± 1.56 b |
|  | Gl | 6.79 ± 0.02 bc | 0.39 ± 0.01 c | 14.37 ± 0.51 abc | 1.62 ± 0.08 b | 8.97 ± 0.74 abc | 173.89 ± 8.49 a | 86.96 ± 11.13 a | 50.69 ± 2.36 b |
|  | Al | 6.84 ± 0.01 ab | 0.32 ± 0 d | 15.67 ± 0.68 a | 1.61 ± 0.08 b | 9.81 ± 0.59 a | 179.02 ± 6.12 a | 82.18 ± 6.86 a | 59.04 ± 2.13 b |
|  | Wh | 6.85 ± 0.01 ab | 0.26 ± 0 e | 15.01 ± 0.44 abc | 1.59 ± 0.12 b | 9.62 ± 0.96 ab | 173.32 ± 4.09 a | 76.28 ± 5.11 a | 52.81 ± 3.73 b |
|  | Ri | 6.7 ± 0.02 d | 0.42 ± 0.01 b | 15.56 ± 1.1 a | 1.75 ± 0.07 ab | 8.97 ± 0.32 abc | 168.20 ± 7.94 a | 76.16 ± 4.56 a | 53.86 ± 1.79 b |
|  | Su | 6.9 ± 0.06 a | 0.2 ± 0 f | 15.38 ± 0.97 ab | 1.92 ± 0.06 a | 8.06 ± 0.27 bcd | 175.42 ± 0.88 a | 82.85 ± 2.56 a | 107.48 ± 6.23 a |

The values are means ± standard deviations (SDs). Different letters indicate significant (Tukey’s HSD, p < 0.05) differences among the values of different treatments at the same sampling time (RSD treatment and two planting seasons).

**Supplementary Table S2** Numbers of bacterial and fungal sequences, ASVs, and coverage in the different soils collected after RSD treatment and two planting seasons.

|  | Samples | | Bacteria | | |  | Fungi | | |
| --- | --- | --- | --- | --- | --- | --- | --- | --- | --- |
|  |  |  | Sequences | ASV_S_ | Coverage |  | Sequences | ASV_S_ | Coverage |
| RSD treatment | Ck | Ck1 | 92386 | 6766 | 0.9562 |  | 40282 | 126 | 0.9712 |
|  |  | Ck2 | 97833 | 7141 | 0.9534 |  | 41651 | 180 | 0.9552 |
|  |  | Ck3 | 84417 | 6458 | 0.9609 |  | 42273 | 153 | 0.9578 |
|  | Et | Et1 | 89928 | 5876 | 0.9653 |  | 39111 | 122 | 0.9829 |
|  |  | Et2 | 95901 | 5991 | 0.9624 |  | 44421 | 139 | 0.9692 |
|  |  | Et3 | 90486 | 5794 | 0.9645 |  | 44207 | 153 | 0.9687 |
|  | Gl | Gl1 | 96341 | 5093 | 0.9688 |  | 48585 | 121 | 0.9685 |
|  |  | Gl2 | 85289 | 4961 | 0.9705 |  | 40024 | 73 | 0.9949 |
|  |  | Gl3 | 82117 | 5557 | 0.9663 |  | 43720 | 127 | 0.9773 |
|  | Al | Al1 | 104329 | 6229 | 0.9583 |  | 43419 | 118 | 0.9831 |
|  |  | Al2 | 104067 | 5715 | 0.9628 |  | 40024 | 131 | 0.9791 |
|  |  | Al3 | 86157 | 5310 | 0.9698 |  | 40919 | 136 | 0.9737 |
|  | Wh | Wh1 | 98596 | 5877 | 0.9631 |  | 32189 | 157 | 0.9688 |
|  |  | Wh2 | 86659 | 5453 | 0.9673 |  | 45941 | 144 | 0.9635 |
|  |  | Wh3 | 92295 | 5978 | 0.9619 |  | 46015 | 177 | 0.9533 |
|  | Ri | Ri1 | 97141 | 6126 | 0.9635 |  | 42445 | 137 | 0.9676 |
|  |  | Ri2 | 101813 | 6360 | 0.9592 |  | 41627 | 163 | 0.9620 |
|  |  | Ri3 | 94321 | 5751 | 0.9656 |  | 47190 | 153 | 0.9591 |
|  | Su | Su1 | 82720 | 5510 | 0.9701 |  | 43620 | 161 | 0.9665 |
|  |  | Su2 | 95384 | 5680 | 0.9669 |  | 44975 | 174 | 0.9566 |
|  |  | Su3 | 85502 | 5429 | 0.9716 |  | 40237 | 158 | 0.9669 |
| First season | Ck | Ck1 | 88588 | 5763 | 0.9644 |  | 38078 | 113 | 0.9791 |
|  |  | Ck2 | 114905 | 6321 | 0.9566 |  | 38064 | 101 | 0.9823 |
|  |  | Ck3 | 81321 | 5481 | 0.9674 |  | 38891 | 92 | 0.9877 |
|  | Et | Et1 | 93383 | 4052 | 0.9774 |  | 34973 | 43 | 0.9913 |
|  |  | Et2 | 88590 | 3865 | 0.9789 |  | 38494 | 40 | 0.9929 |
|  |  | Et3 | 97711 | 4232 | 0.9761 |  | 38401 | 34 | 0.9915 |
|  | Gl | Gl1 | 104426 | 4647 | 0.9717 |  | 36126 | 66 | 0.9873 |
|  |  | Gl2 | 109433 | 4600 | 0.9718 |  | 35298 | 44 | 0.9993 |
|  |  | Gl3 | 106573 | 3881 | 0.9797 |  | 36481 | 46 | 0.9946 |
|  | Al | Al1 | 81935 | 4304 | 0.9771 |  | 34486 | 77 | 0.9929 |
|  |  | Al2 | 101339 | 4599 | 0.9736 |  | 33373 | 57 | 0.9983 |
|  |  | Al3 | 95131 | 4446 | 0.9750 |  | 37970 | 72 | 0.9945 |
|  | Wh | Wh1 | 85374 | 4493 | 0.9745 |  | 32907 | 87 | 0.9828 |
|  |  | Wh2 | 89149 | 4568 | 0.9745 |  | 35308 | 78 | 0.9925 |
|  |  | Wh3 | 104172 | 5028 | 0.9682 |  | 35808 | 52 | 0.9998 |
|  | Ri | Ri1 | 70999 | 4712 | 0.9762 |  | 38980 | 105 | 0.9760 |
|  |  | Ri2 | 79294 | 4985 | 0.9723 |  | 40659 | 80 | 0.9864 |
|  |  | Ri3 | 90861 | 5744 | 0.9647 |  | 41602 | 95 | 0.9796 |
|  | Su | Su1 | 76361 | 5710 | 0.9672 |  | 25462 | 59 | 0.9880 |
|  |  | Su2 | 101222 | 5898 | 0.9624 |  | 37167 | 104 | 0.9832 |
|  |  | Su3 | 103632 | 6229 | 0.9589 |  | 33426 | 110 | 0.9738 |
| Second season | Ck | Ck1 | 76996 | 5314 | 0.9716 |  | 89093 | 115 | 0.9624 |
|  |  | Ck2 | 71188 | 5155 | 0.9741 |  | 102642 | 123 | 0.9705 |
|  |  | Ck3 | 65241 | 4978 | 0.9767 |  | 95116 | 117 | 0.9629 |
|  | Et | Et1 | 57622 | 4235 | 0.9826 |  | 89355 | 75 | 0.9770 |
|  |  | Et2 | 70663 | 4551 | 0.9785 |  | 89564 | 62 | 0.9791 |
|  |  | Et3 | 66889 | 4315 | 0.9801 |  | 96084 | 72 | 0.9823 |
|  | Gl | Gl1 | 68252 | 4057 | 0.9820 |  | 87998 | 84 | 0.9784 |
|  |  | Gl2 | 70128 | 4192 | 0.9804 |  | 76378 | 89 | 0.9855 |
|  |  | Gl3 | 79594 | 4819 | 0.9743 |  | 81503 | 96 | 0.9764 |
|  | Al | Al1 | 65591 | 4524 | 0.9785 |  | 86635 | 100 | 0.9811 |
|  |  | Al2 | 72001 | 4364 | 0.9788 |  | 87105 | 116 | 0.9728 |
|  |  | Al3 | 72752 | 4463 | 0.9774 |  | 89613 | 109 | 0.9798 |
|  | Wh | Wh1 | 76361 | 4863 | 0.9738 |  | 95878 | 101 | 0.9838 |
|  |  | Wh2 | 53497 | 3966 | 0.9833 |  | 92590 | 125 | 0.9723 |
|  |  | Wh3 | 71303 | 4792 | 0.9756 |  | 97844 | 109 | 0.9795 |
|  | Ri | Ri1 | 58224 | 4486 | 0.9803 |  | 96765 | 123 | 0.9679 |
|  |  | Ri2 | 69323 | 5042 | 0.9747 |  | 89143 | 90 | 0.9741 |
|  |  | Ri3 | 55142 | 4377 | 0.9817 |  | 90248 | 132 | 0.9627 |
|  | Su | Su1 | 64165 | 4844 | 0.9777 |  | 103680 | 135 | 0.9729 |
|  |  | Su2 | 58837 | 4682 | 0.9796 |  | 83992 | 133 | 0.9683 |
|  |  | Su3 | 70536 | 5185 | 0.9744 |  | 84762 | 140 | 0.9574 |

**Supplementary Table S3** Alpha diversity indices for bacterial and fungal communities in different soils collected after RSD treatment and two planting seasons.

|  | Samples | Bacteria | |  | Fungi | |
| --- | --- | --- | --- | --- | --- | --- |
|  |  | Shannon | Chao1 |  | Shannon | Chao1 |
| RSD treatment | Ck | 11.29 ± 0.08 a | 8495.32 ± 531.48 a |  | 5.76 ± 0.33 ab | 194.23 ± 28.63 ab |
|  | Et | 10.89 ± 0.02 bc | 7300.2 ± 175.72 ab |  | 5.75 ± 0.07 ab | 160.28 ± 24.9 ab |
|  | Gl | 10.18 ± 0.1 e | 6340.35 ± 340.64 b |  | 4.89 ± 0.46 b | 125.5 ± 44.64 b |
|  | Al | 10.67 ± 0.11 cd | 7257.26 ± 764.76 ab |  | 5.58 ± 0.23 ab | 143.89 ± 15.14 ab |
|  | Wh | 10.76 ± 0.13 bcd | 7163.59 ± 429.92 b |  | 5.6 ± 0.58 ab | 191.29 ± 25.5 ab |
|  | Ri | 10.94 ± 0.07 b | 7561.94 ± 429.02 ab |  | 5.61 ± 0.2 ab | 188.7 ± 16.33 ab |
|  | Su | 10.56 ± 0.08 d | 6615.73 ± 310.75 b |  | 6.16 ± 0.04 a | 200.52 ± 16.41 a |
| First season | Ck | 10.95 ± 0.08 a | 7430.54 ± 777.58 a |  | 5.28 ± 0.08 a | 116.94 ± 13.96 a |
|  | Et | 9.38 ± 0.13 d | 4891.61 ± 251.38 c |  | 2.42 ± 0.33 c | 45.79 ± 4.91 c |
|  | Gl | 9.93 ± 0.07 c | 5381.88 ± 615.04 c |  | 4.1 ± 0.11 b | 56.04 ± 15.9 bc |
|  | Al | 10.39 ± 0.04 b | 5405.25 ± 237.66 c |  | 4.83 ± 0.19 ab | 69.91 ± 10.85 abc |
|  | Wh | 10.49 ± 0.08 b | 5824.87 ± 525.32 bc |  | 4.88 ± 0.17 ab | 79.67 ± 27.41 abc |
|  | Ri | 10.81 ± 0.14 a | 6220.13 ± 836.65 bc |  | 4.56 ± 0.2 ab | 109.49 ± 15.26 ab |
|  | Su | 11.05 ± 0.06 a | 7435.38 ± 543.34 a |  | 4.73 ± 0.77 ab | 110.48 ± 35.04 ab |
| Second season | Ck | 10.8 ± 0.05 a | 5949.82 ± 313.96 a |  | 4.95 ± 0.7 a | 164.61 ± 13.51 a |
|  | Et | 10.23 ± 0.04 cd | 4898.71 ± 259.72 b |  | 3.4 ± 0.29 b | 96.85 ± 9.41 c |
|  | Gl | 10.17 ± 0.11 d | 5002.27 ± 568.89 b |  | 4.84 ± 0.11 a | 109.93 ± 11.83 bc |
|  | Al | 10.45 ± 0.02 bc | 5145.6 ± 96.86 ab |  | 4.85 ± 0.06 a | 125.29 ± 14.82 abc |
|  | Wh | 10.51 ± 0.15 b | 5256.71 ± 705.11 ab |  | 5.45 ± 0.1 a | 129.21 ± 20.81 abc |
|  | Ri | 10.63 ± 0.09 ab | 5246.11 ± 501.2 ab |  | 4.82 ± 0.22 a | 152.6 ± 26.6 ab |
|  | Su | 10.8 ± 0.06 a | 5565.5 ± 352.63 ab |  | 5.29 ± 0.02 a | 169.62 ± 22.12 a |

The values are means ± standard deviations (SDs). Different letters indicate significant (Tukey’s HSD, *P* < 0.05) differences among the values of different treatments at the same sampling time (RSD treatment and two planting seasons).

**Supplementary Table S4** Microbial community dissimilarity comparison among treatments using two nonparametric statistical methods of analysis of similarity (ANOSIM) and non-parametric multivariate analysis of variance (PERMANOVA).

| Treatments | Adonis | |  | ANOSIM | |
| --- | --- | --- | --- | --- | --- |
|  | *F* | *p* |  | *R* | *p* |
| *Simple carbon vs Complex carbon after RSD treatment* | |  |  |  |  |
| Bacteria | 4.231 | 0.001 |  | 0.478 | 0.002 |
| Fungi | 2.905 | 0.002 |  | 0.391 | 0.004 |
| *Simple carbon vs Complex carbon after First season* | |  |  |  |  |
| Bacteria | 10.392 | 0.001 |  | 0.877 | 0.001 |
| Fungi | 6.476 | 0.001 |  | 0.821 | 0.001 |
| *Simple carbon vs Complex carbon after Second season* | |  |  |  |  |
| Bacteria | 7.547 | 0.001 |  | 0.862 | 0.001 |
| Fungi | 7.625 | 0.001 |  | 0.898 | 0.001 |
| *RSD treatment vs First season* |  |  |  |  |  |
| Bacteria | 13.570 | 0.001 |  | 0.769 | 0.001 |
| Fungi | 17.976 | 0.001 |  | 0.858 | 0.001 |
| *First season vs Second season* |  |  |  |  |  |
| Bacteria | 6.322 | 0.001 |  | 0.330 | 0.001 |
| Fungi | 10.472 | 0.001 |  | 0.662 | 0.001 |
| *RSD treatment vs Second season* |  |  |  |  |  |
| Bacteria | 18.061 | 0.001 |  | 0.959 | 0.001 |
| Fungi | 12.852 | 0.001 |  | 0.687 | 0.001 |
| *RSD treatment vs First season vs Second season* | |  |  |  |  |
| Bacteria | 12.617 | 0.001 |  | 0.678 | 0.001 |
| Fungi | 13.612 | 0.001 |  | 0.715 | 0.001 |

**Supplementary Table S5** Relationships between carbon source, crop cultivation, soil chemical property, and microbial community dissimilarities analyzed using Mantel tests.

|  | Bacteria | |  | Fungi | |
| --- | --- | --- | --- | --- | --- |
|  | *r* | *p* |  | *r* | *p* |
| Carbon source property | 0.151 | 0.045 |  | 0.369 | 0.001 |
| Crop cultivation | 0.731 | 0.001 |  | 0.340 | 0.001 |
| Soil chemical property | 0.530 | 0.001 |  | 0.128 | 0.097 |

**Supplementary Table S6** Pearson's correlation coefficients of bacterial and fungal ASVs and impact factors based on Mantel tests.

|  | Bacteria | |  | Fungi | |
| --- | --- | --- | --- | --- | --- |
|  | *r* | *p* |  | *r* | *p* |
| Org TOC | -0.011 | 0.486 |  | **0.187** | **0.025** |
| Org TN | -0.026 | 0.553 |  | -0.069 | 0.724 |
| Org C/N | **0.250** | **0.006** |  | **0.401** | **0.002** |
| Org EOC | **0.204** | **0.034** |  | **0.424** | **0.001** |
| Crop cultivation | **0.721** | **0.001** |  | **0.405** | **0.001** |
| pH | **0.297** | **0.013** |  | 0.174 | 0.104 |
| EC | **0.245** | **0.045** |  | -0.047 | 0.578 |
| TOC | 0.070 | 0.328 |  | 0.206 | 0.075 |
| TN | 0.053 | 0.310 |  | 0.070 | 0.251 |
| C/N | -0.029 | 0.532 |  | 0.106 | 0.196 |
| AN | **0.302** | **0.021** |  | -0.078 | 0.695 |
| AP | **0.555** | **0.001** |  | **0.204** | **0.027** |
| AK | **0.497** | **0.001** |  | 0.169 | 0.064 |

The impact factors’ abbreviations are defined in Table 1 and Fig.1.

**Supplementary Table S7** Redundancy analysis (RDA) of all assigned bacterial and fungal ASVs and impact factors.

|  | Bacteria | |  | Fungi | |
| --- | --- | --- | --- | --- | --- |
|  | *r^2^* | *p* |  | *r^2^* | *p* |
| Org TOC | 0.312 | 0.056 |  | **0.419** | **0.011** |
| Org TN | 0.159 | 0.274 |  | 0.065 | 0.592 |
| Org C/N | **0.500** | **0.011** |  | **0.520** | **0.005** |
| Org EOC | **0.504** | **0.013** |  | **0.635** | **0.001** |
| Crop cultivation | **0.937** | **0.001** |  | **0.540** | **0.006** |
| pH | 0.200 | 0.162 |  | **0.436** | **0.015** |
| EC | 0.221 | 0.151 |  | 0.214 | 0.176 |
| TOC | 0.320 | 0.054 |  | 0.219 | 0.153 |
| TN | 0.115 | 0.396 |  | 0.299 | 0.073 |
| C/N | 0.045 | 0.724 |  | 0.199 | 0.196 |
| AN | 0.383 | 0.032 |  | 0.283 | 0.085 |
| AP | **0.875** | **0.001** |  | 0.249 | 0.094 |
| AK | **0.763** | **0.001** |  | 0.298 | 0.062 |

The impact factors’ abbreviations are defined in Table 1 and Fig.1.

**Supplementary Table S8** Spearman's correlation coefficients of carbon source properties and RA of *Fusarium* in soils collected after RSD treatment.

|  | **Org TOC (g kg^-1^)** | **Org TN (g kg^-1^)** | **Org C/N** | **Org EOC (g kg^-1^)** |
| --- | --- | --- | --- | --- |
| **RA of *Fusarium*** | -0.714 | 0.522 | -0.522 | **-0.943^**^** |

^*^, *p* < 0.05; ^**^, *p* < 0.01; ^***^, *p* < 0.001.

**Supplementary Table S9** Spearman's correlation coefficients of carbon source properties and crop yield of the complex-carbon soils after the first season.

|  | **Org TOC (g kg^-1^)** | **Org TN (g kg^-1^)** | **Org C/N** | **Org EOC (g kg^-1^)** |
| --- | --- | --- | --- | --- |
| **Crop yield** | -0.200 | **1.000^**^** | **-1.000^**^** | 0.600 |

^*^, *p* < 0.05; ^**^, *p* < 0.01; ^***^, *p* < 0.001.


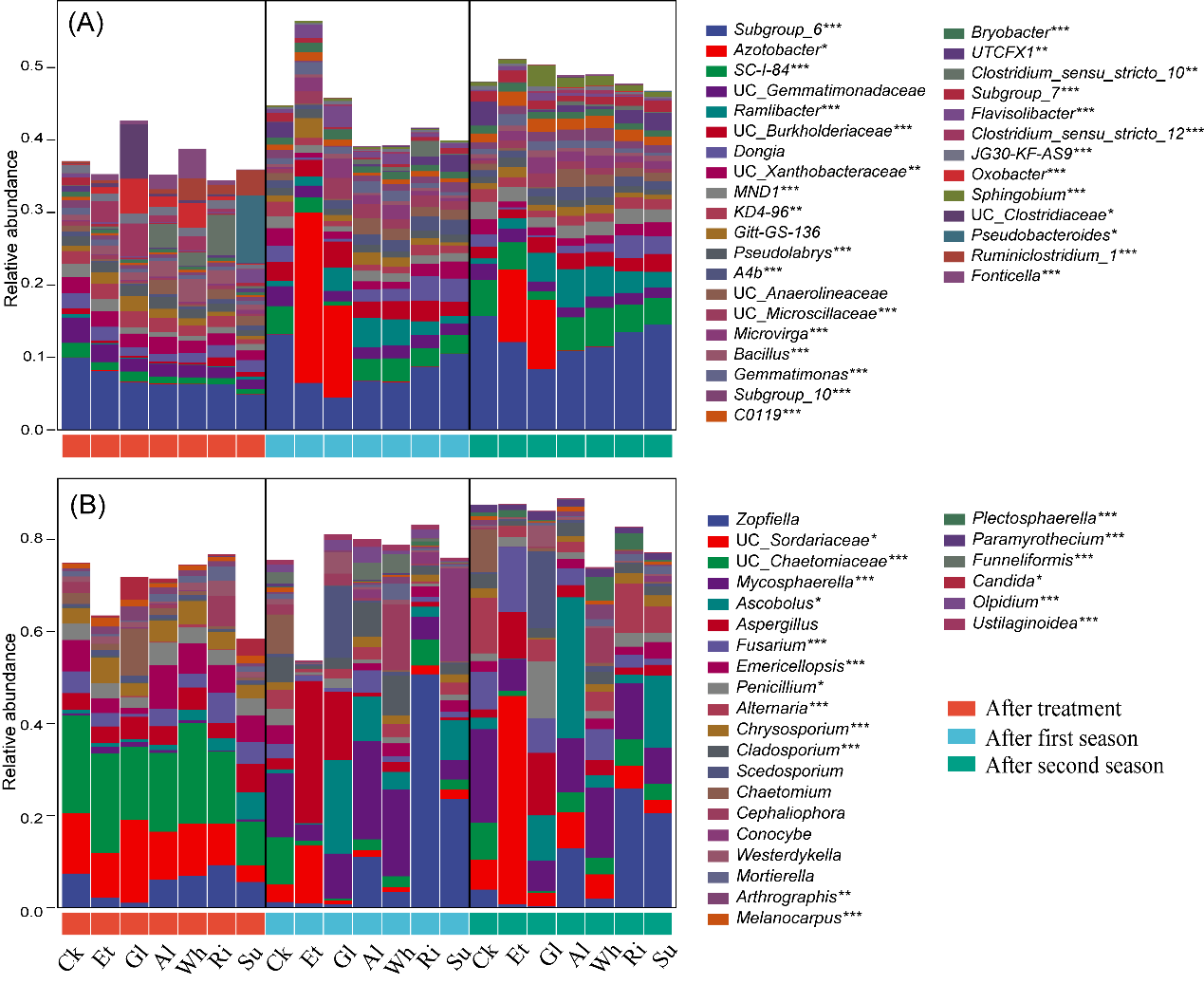
 **Supplementary Figure S1** Relative abundances of bacterial (A) and fungal (B) genera in different soils collected after RSD treatment and two planting seasons. Only bacterial and fungal genera with average relative abundance > 1% after RSD treatment, first season, or second season are shown. Letters after "UC_" indicate the most detailed classification for unclassified genera. “^*^” *p* < 0.05, “^**^” *p* < 0.01, and “^***^” *p* < 0.001 represent significant differences of bacterial or fungal genera among soils collected after RSD treatment and two planting seasons according to Tukey’s HSD. Treatment abbreviations are defined in Table 1.


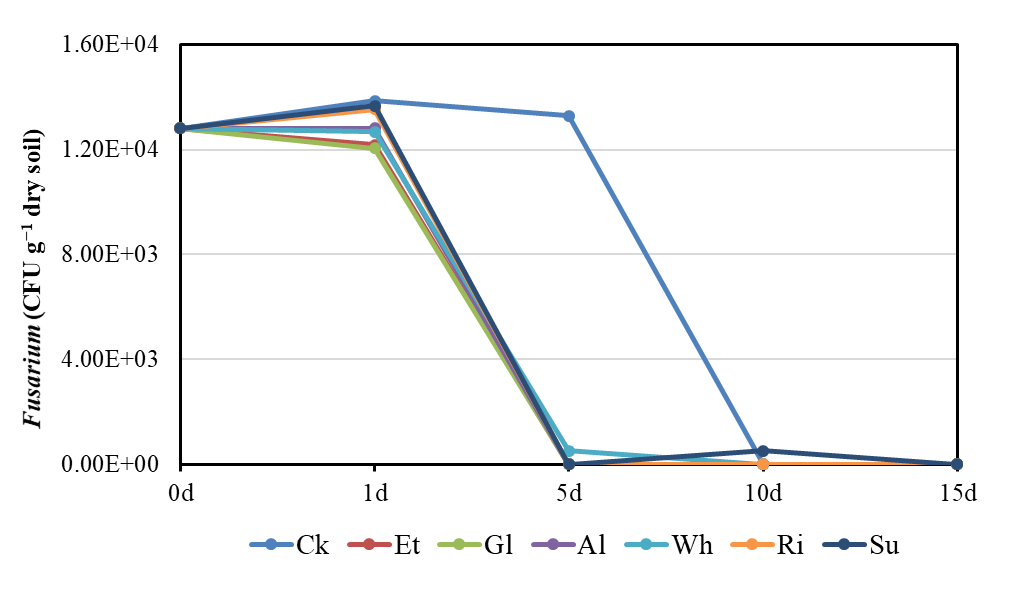


**Supplementary Figure S2** The changes of cultivable *Fusarium* number in different soils during the 15-day RSD treatment.


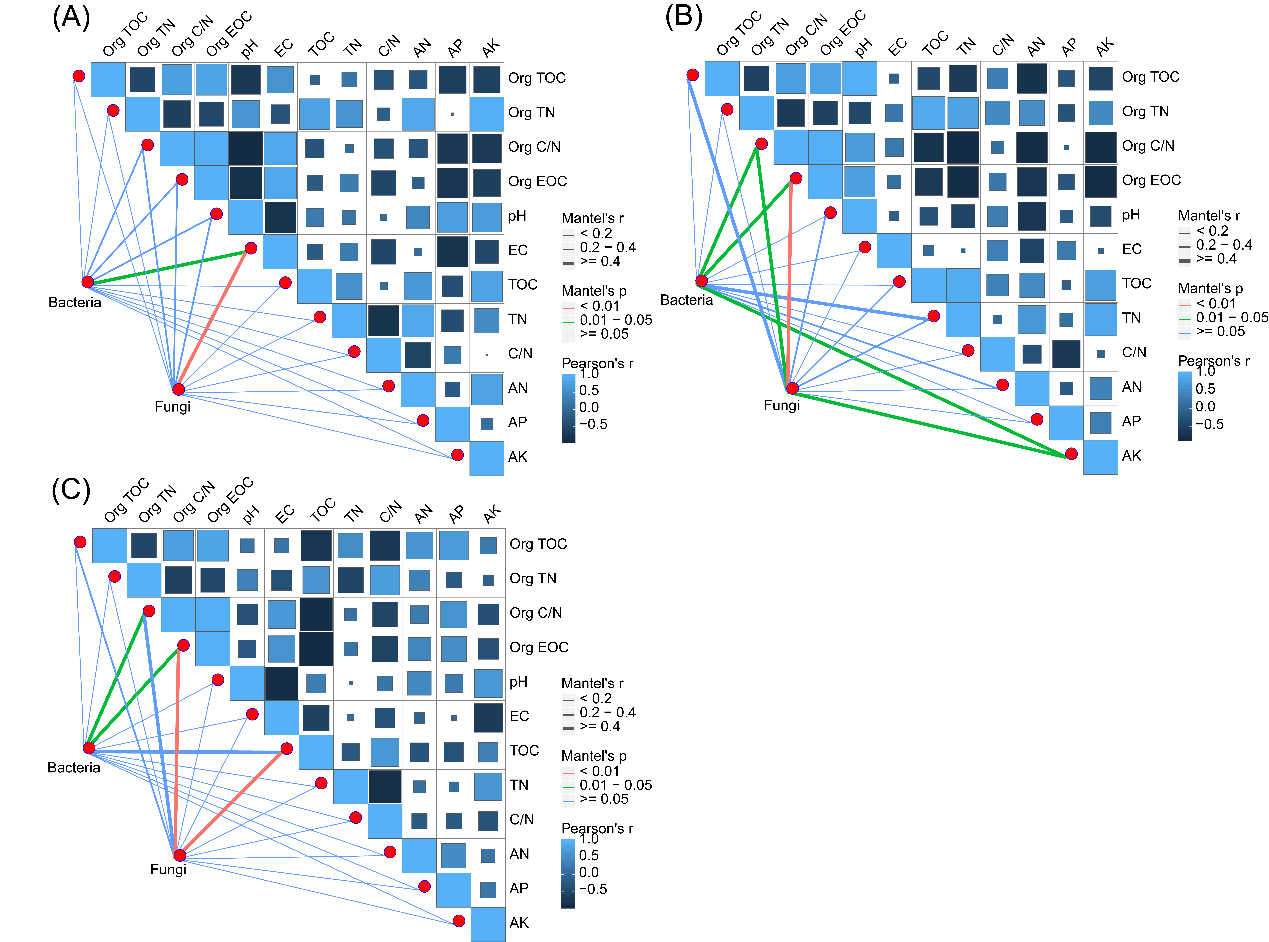


**Supplementary Figure S3** Pearson's correlation coefficients of bacterial and fungal ASVs in soils collected after RSD treatment (A), first season (B), and second season (C) and impact factors based on Mantel tests.

**
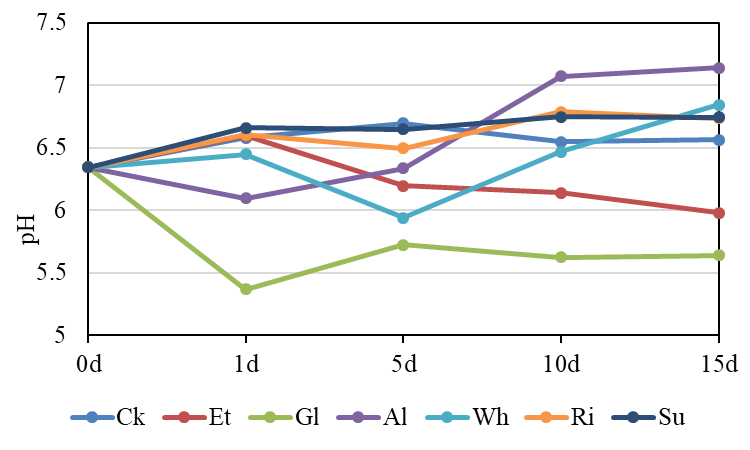
**

**Supplementary Figure S4** The changes of pH values in different soils during the 15-day RSD treatment.
